# Essential Oil of the Plants Growing in the Brazilian Amazon: Chemical Composition, Antioxidants, and Biological Applications

**DOI:** 10.3390/molecules27144373

**Published:** 2022-07-08

**Authors:** Oberdan Oliveira Ferreira, Jorddy Neves Cruz, Ângelo Antônio Barbosa de Moraes, Celeste de Jesus Pereira Franco, Rafael Rodrigues Lima, Taina Oliveira dos Anjos, Giovanna Moraes Siqueira, Lidiane Diniz do Nascimento, Márcia Moraes Cascaes, Mozaniel Santana de Oliveira, Eloisa Helena de Aguiar Andrade

**Affiliations:** 1 Program of Post-Graduation in Biodiversity and Biotecnology (Bionorte), Federal University of Pará, Belem 66075-110, PA, Brazil; oberdan@museu-goeldi.br (O.O.F.); eloisa@museu-goeldi.br (E.H.d.A.A.); 2 Adolpho Ducke Laboratory, Botany Coordination, Museu Paraense Emílio Goeldi, Av. Perimetral, 1901-Terra Firme, Belem 66077-830, PA, Brazil; angeloquimica17@gmail.com (Â.A.B.d.M.); cleste.frango12@gmail.com (C.d.J.P.F.); giovannamsiqueiraa@gmail.com (G.M.S.); lidianenascimento@museu-goeldi.br (L.D.d.N.); 3 Laboratory of Functional and Structural Biology, Institute of Biological Sciences, Federal University of Pará, Belem 66075-110, PA, Brazil; jorddynevescruz@gmail.com (J.N.C.); rafalima@ufpa.br (R.R.L.); 4 Program of Post-Graduation in Botany, Paraense Emílio Goeldi Museum, Av. Perimetral, 1901-Terra Firme, Belem 66077-830, PA, Brazil; tainadosanjoscb@gmail.com; 5 Program of Post-Graduation in Chemistry, Federal University of Pará, Belem 66075-110, PA, Brazil; cascaesmm@gmail.com

**Keywords:** species of Brazil, essential oils, bioactive compounds, biological activities

## Abstract

Essential oils are biosynthesized in the secondary metabolism of plants, and in their chemical composition, they can be identified different classes of compounds with potential antioxidant and biological applications. Over the years in the Amazon, several species of aromatic plants were discovered and used in traditional medicine. The literature has shown that essential oils extracted from amazon species have several biological activities, such as antioxidant, antibacterial, antifungal, cytotoxic, and antiprotozoal activities. These activities are related to the diversified chemical composition found in essential oils that, by synergism, favors its pharmacological action. In light of this vital importance, this study aimed at performing a review of the literature with particular emphasis on the chemical composition and biological activities in studies conducted with species collected in the Amazon, taking into consideration in particular the last 10 years of collection and research.

## 1. Introduction

Brazil has the world’s highest plant diversity. It houses more than 46,000 species of plants, algae, and fungi, and most of this biodiversity is found in the Amazon [1,2]. This biome occupies 5 million km^2^ of the territory, corresponding to 60% of the entire national territory. Such areas include the Brazilian Amazon, which accounts for 51% of all tropical plant species. The Brazilian Amazon forest accounts for approximately 26% of the remaining tropical rainforests on Earth [3,4].

Typifying this exuberance, 12 families that provide essential oil are predominant in the Amazon region (in descending order): Piperaceae, Asteraceae, Myrtaceae, Lamiaceae, Annonaceae, Lauraceae, Euphorbiaceae, Verbenaceae, Scrophulariaceae, Anacardiaceae, Burseraceae, and Rutaceae [5,6].

Essential oils are volatile, with a strong smell and taste derived from the secondary metabolites of the plants. Essential oils can be extracted from the roots, stems, leaves, and flowers by steam distillation, hydrodistillation, and squeezing citrus fruit pericarps. The terminology “oil” is closely related to the physicochemical characteristics of these substances, as they are liquids at room temperature [7,8].

The biological activity of essential oils is due to the diversity of chemical components in these volatile oils. These properties include antibacterial, antifungal, and antioxidant activities [9,10,11,12]. Essential oils can also be used as raw materials for products such as cosmetics and perfumes, or in pharmaceutical industries to obtain structural derivatives (plant products) in addition to horticulture [7,13].

Although essential oils have several potential applications, many aromatic plants in the Amazon ecosystem are under constant environmental pressure, as this region undergoes increasing fires, deforestation, and unsustainable forest exploitation [5].

Although Brazil is still the largest natural angiosperm bank in the world and these aromatic plants have the potential for varied uses, part of this exuberance was lost long before scientific knowledge was gained [3,14]. Therefore, efforts and resources must be invested to acquire a greater awareness of the diversity and value of the plants that remain in the Amazon region.

Therefore, this chapter provides a bibliographic survey of scientific articles reporting the chemical composition and antioxidant and biological activities of species collected in the Amazon, taking into consideration the last ten years.

## 2. Chemical Composition of the Essential Oils of the Amazon

Table 1 shows the major chemical components found in the essential oils of the species from the Amazon region.

**Table 1:** Major chemical constituents (≥3.00%) found in the essential oils of the Amazon.

Species	Family	Extraction Method	Compounds	References
*Anaxagorea brevipes* (leaves)	Annonaceae	HD	β-eudesmol (13.16%), α-eudesmol (13.05%), γ-eudesmol (7.54%), guaiol (5.12%), caryophyllene oxide (4.18%) and β-bisabolene (4.10%)	[15]
*Aniba duckei* (Synonym: *A. rosaeodora*) (leaves and thin branches)	Lauraceae	HD	linalool (89.34%)	[16]
*A. parviflora* (Aerial parts)	Lauraceae	HD	linalool (45.0%)	[17]
*A. parviflora* (branches)	Lauraceae	HD	γ-eudesmol (16.80%), (*E*)-caryophyllene (15.70%), linalool (12.40%), β-phellandrene (6.7%), and bicyclogermacrene (6.00%)	[18]
*A. parviflora* (leaves)	Lauraceae	HD	β-phellandrene (15.10%), linalool (14.10%) and γ-eudesmol (12.90%).	[18]
*A. rosaeodora* (Aerial parts)	Lauraceae	HD	linalool (88.60%)	[17]
*A. rosaeodora* (Aerial parts)	Lauraceae	HD	linalool (93.60%)	[19]
*Annona exsucca* (Dry leaves)	Annonaceae	HD	(*E*)-caryophyllene (31.26%), linalool (10.80%), β-elemene (10.30%), germacrene D (10.28%), bicyclogermacrene (9.84%)	[20]
*Bauhinia ungulata* (leaves)	Fabaceae	HD	(*E*)-caryophyllene (15.9%), caryophyllene oxide (9.2%) α-humulene (8.1%) and *epi*-γ-eudesmol (7.5%)	[21]
*Bocageopsis pleiosperma* (Barks)	Annonaceae	HD	β-bisabolene (38.53%), δ-cadinene (7.55%), β-selinene (6.46%) and α-selinene (5.18%)	[22]
*B. pleiosperma* (leaves)	Annonaceae	HD	β-bisabolene (55.77%), (*E*)-α-bergamotene (6.94%) and β-farnesene (*E*) (6.05%)	[22]
*B. pleiosperma* (twigs)	Annonaceae	HD	β-bisabolene (34.37%), cryptomerione (9,60%) and (2*Z*, 6*Z*)-farnesol (7,20%),	[22]
*B. multiflora* (Leaves)	Annonaceae	HD	spathulenol (20.30%) and β-bisabolene (11.90%)	[23]
*B. multiflora* (Aerial parts)	Annonaceae	HD	*cis*-linalool oxide (33.10%) and 1-epi-cubenol (16.60%).	[24]
*B. multiflora* (fresh leaves)	Annonaceae	HD	spathulenol (13.00–16.20%), β-bisabolene (13.20–13.80%) and caryophyllene oxide (10.70–12.00%)	[25]
*Copaifera multijuga* (resin)	Fabaceae	Perforation in the trunk of the species	(*E*)-caryophyllene (57.29%), caryophyllene oxide (10.34%) and α-humulene (9.11%)	[26]
*Croton cajucara* (leaves)	Euphorbiaceae	HD	7-hydroxycalamenene	[27]
*Duguetia quitarensis* (Aerial parts)	Annonaceae	HD	4-heptanol (33.80%), α-thujene (18.40%) and (*E*)-caryophyllene (14.40%)	[24]
*Endlicheria arenosa* (Leaves)	Lauraceae	HD	bicyclogermacrene (42.20%) and (*E*)-caryophyllene (10.10%).	[28]
*E. arenosa* (Twigs)	Lauraceae	HD	limonene (33.20%) and terpinen-4-ol (15.60%)	[28]
*Ephedranthus amazonicus* (Leaves)	Annonaceae	HD	spathulenol (16.90%) and humulene epoxide II (16.30%)	[23]
*Eugenia cuspidifolia* (Dry leaves)	Myrtaceae	HD	caryophyllene oxide (57.46%) and α-copaene (3.75%)	[29]
*E. egensis* (Aerial parts)	Myrtaceae	HD	5-hydroxy-(*Z*)-calamenene (35.80%), (*E*)-caryophyllene (8.90%) and (*E*)-cadina-1,4-diene (6.30%)	[30]
*E. flavescens* (Aerial parts)	Myrtaceae	HD	(*E*)-γ-bisabolene (35.00%) and β-bisabolene (34.70%)	[30]
*E. patrisii* (Aerial parts)	Myrtaceae	HD	(2*E*,6*E*)-Farnesol (34.50%) and (2*E*,6*Z*)-Farnesol (23.20%).	[30]
*E. patrisii* (Dry leaves)	Myrtaceae	HD	May: germacrene D (20.03%), bicyclogermacrene (11.82%) and (*E*)-caryophyllene (11.04%) September: γ-elemene (25.89%), (*E*)-caryophyllene (10.76%) and germacrene B (8.11%)	[31]
*E. patrisii* (Leaves)	Myrtaceae	HD	(*E*)-caryophyllene (32.00%) and bicyclogermacrene (10.00%)	[32]
*E. piauhiensis* (dry leaves)	Myrtaceae	HD	γ-elemene (17.48%), (*E*)- caryophyllene (16.46%) and bicyclogermacrene (8.11%)	[33]
*E. polystachya* (Aerial parts)	Myrtaceae	HD	germacrene D (18.40%), ishwarane (15.70%) and 7-*epi*-α-selinene (7.50%)	[30]
*E. punicifolia* (Dry leaves)	Myrtaceae	HD	May: β-elemene (25.12%), (*E*)-caryophyllene (13.11%), bicyclogermacrene (9.88%) and selin-11-en-4α-ol (9.16%) September: (*E*)-caryophyllene (11.47%), β-pinene (5.86%), bicyclogermacrene (5.86%), and γ-muurolene (5.55%)	[31]
*E. stipitata* (Leaves)	Myrtaceae	HD	germacrene D (11.80%) and *Z*-α-bisabolene (8.38%).	[32]
*E. uniflora* (leaves)	Myrtaceae	HD	Curzerene (34.40—53.10%)	[34]
*E. tapacumensis* (Dry leaves)	Myrtaceae	HD	caryophyllene oxide (55.95%) and α-copaene (13.67%)	[29]
*Fusaea longifolia* (Aerial parts)	Annonaceae	HD	β-selinene (19.30%), *cis*-β-guaiene (18.30%), (*Z*)-α-bisabolene (12.00%) and (*E*)-caryophyllene (7.10%)	[24]
*Guatteria blepharophylla* (Leaves)	Annonaceae	HD	caryophyllene oxide (55.70%).	[23]
*G. friesiana* (dry leaves)	Annonaceae	HD	β-eudesmol (51.92 ± 9.15%), γ-eudesmol (18.91 ± 5.41%) and α-eudesmol (12.56 ± 2.80%)	[35]
*G. megalophylla*	Annonaceae	HD	spathulenol (27.76%), γ-muurolene (14.34%), bicyclogermacrene (10.47%) and β-elemene (7.48%)	[36]
*G. pogonopus* (dry leaves)	Annonaceae	HD	spathulenol (24.80 ± 11.38%), γ-amorphene (14.72 ± 3.37%) and germacrene D (11.75 ± 6.33%).	[35]
*G. punctata* (Aerial parts)	Annonaceae	HD	germacrene D (19.80%), (*E*)-nerolidol (9.90%) and (*E*)-caryophyllene (8.40%).	[24]
*Hedychium coronarium* (Rhizome)	Zingiberaceae	HD	eucalyptol (33.70%), β-pinene (30.00%) and α-pinene (10.00%)	[37]
*Ipomea setifera* (Dry leaves)	Convolvulaceae	SD	(*E*)-caryophyllene (36.70%) and β-elemene (20.49%)	[38]
*I. asarifolia* (Dry leaves)	Convolvulaceae	SD	phytol derivade (10.67–35.49%) and (*E*)-caryophyllene (15.93–19.93%)	[38]
*Iryanthera polyneura* (Leaves)	Myristicaceae	HD	spathulenol (6.42 ± 1.02%), α-cadinol (5.82 ± 0.40%) and τ-muurolol (5.24 ± 0.03%).	[39]
*Lippia gracilis* (dry leaves)	Verbenaceae	HD	limonene (56.16%), geraniol (12.09%) and β-myrcene (6.22%).	[33]
*L. origanoides* (aerial parts)	Verbenaceae	HD	Carvacrol (37.12%), *p*-cymene (11.64%) and thymol (7.83%)	[40]
*L. origanoides* (leaves)	Verbenaceae	HD	carvacrol (48.31%), *p*-cymene (9.11%), thymol (8.78%), (*E*)-caryophyllene (6.74%) and 2,5-dimethoxyacetophenone (6.63%)	[41]
*L. thymoides* (Fresh and Dry Leaves)	Verbenaceae	HD	thymol (59.29–62.78%), *p*-cymene (2.97–8.97%), (*E*)-caryophyllene (5.21–8.84%) and thymyl acetate (4.92–7.22%).	[42]
*L. thymoides* (Freash and Dry leaves)	Verbenaceae	HD	thymol (58.90–66.33%), thymol acetate (7.49–8.10%), γ-terpinene (7.58–9.36%) and *p*-cymene (5.30–8.36%).	[43]
*L. thymoides* (Freash and Dry flowers)	Verbenaceae	HD	thymol (37.86–48.04%), thymol acetate (21.44–33.81), γ-terpinene (0.15–15.06%) and *p*-cymene (0.07–7.18%)	[43]
*L. thymoides* (Freash and Dry branches)	Verbenaceae	HD	thymol (63.59–66.20%), thymol acetate (5.07–5.96%) γ-terpinene (3.39–9.36%) and *p*-cymene (3.27–3.35%)	[43]
*L. thymoides* (Freash and Dry roots)	Verbenaceae	HD	(11*Z*)-11-hexadecenoic acid (38–02-40.92%), (9*Z*)-octadecenoic acid (27.40–28.21%) and thymol (19.34–22.18%)	[43]
*Mentha piperita* (Dry leaves)	Lamiaceae	HD	linalool (51.80%) and epoxyocimene (19.30%).	[44]
*Mesosphaerum suaveolens* (aerial parts)	Lamiaceae	HD	eucalyptol (30.15–64.44%), linalool (0.00–12.85%), β-pinene (3.27–9.04%) and sabinene (0.00–8.58%)	[45]
*Myrcia erythroxylon* (Dry leaves)	Myrtaceae	HD	α-humulene (26.79%), bicyclogermacrene (13.26%) and (*E*)-caryophyllene (10.55%)	[33]
*M. splendens* (Leaves)	Myrtaceae	HD	(*E*)-caryophyllene (45.80%)	[32]
*M. splendens* (Leaves)	Myrtaceae	HD	(*E*)-caryophyllene (36.23%), *trans*-γ-bisabolene (10.04%), *cis*-γ-bisabolene (8.33%) and *trans*-β-farnesene (7.81%)	[46]
*M. sylvatica* (Leaves)	Myrtaceae	HD	germacrene B (24.50%) and γ-elemene (12.50%)	[32]
*M. sylvatica* (Fresh leaves)	Myrtaceae	HD	1-*epi*-cubenol (9.90%), cadalene (7.20%), β-selinene (7.00%), β-calacorene (5.40%), *cis*-calamenene (4.80%), muskatone (4.40%), δ-cadinene (4.20%), cubenol (4.20%) and *ar*-curcumene (1.90%)	[10]
*M. sylvatica* (Dried Leaves)	Myrtaceae	HD	*ar*-curcumene (7.60%), 1-*epi*-cubenol (6.90%), β-selinene (6.00%), cadalene (5.80%), β-calacorene (5.50%), *cis*-calamenene (5.20%), arturmerol (4.90%), δ-cadineno (4.20%), cubenol (4.20%) and muskatone (3.40%).	[10]
*M. tomentosa* (Dry leaves)	Myrtaceae	HD	May: γ-elemene (12.52%), germacrene D (11.45%) and (*E*)-caryophyllene (10.22%) September: spathulenol (40.70%), zingiberene (9.58%) and γ-elemene (6.89%)	[31]
*Nectandra cuspidata* (*Leaves*)	Lauraceae	HD	(*E*)-caryophyllene (26.90%) and bicyclogermacrene (16.00%)	[47]
*N. puberula* (Leaves)	Lauraceae	HD	apiole (22.20%), (*E*)-caryophyllene (15.10%) and β-pinene (13.30%).	[47]
*N. puberula* (branches)	Lauraceae	HD	apiole (28.10%), pogostol (19.80%) and viridiflorol (11.20%)	[47]
*Ocimum campechianum* (leaves and stems)	Lamiaceae	HD	methyleugenol (80.00– 87.00%)	[48]
*O. campechianum* (inflorescences)	Lamiaceae	HD	methyleugenol (75.30–83.50%)	[48]
*O. canum* (dry leaves)	Lamiaceae	HD	thymol (42.15%), *p*-cymene (21.17%) and γ-terpinene (19.81%)	[49]
*Ocotea caniculata* (leaves)	Lauraceae	HD	β-selinene (20.30%), β-caryophyllene (18.90%) and 7-epi-α-selinene (14.30%)	[50]
*O. caniculata* (branches)	Lauraceae	HD	selin-11-en-4-α-ol (20.60%), β-selinene (12.10%) and 7-epi-α-selinene (9.00%)	[50]
*O. caudata* (leaves)	Lauraceae	HD	bicyclogermacrene (29.60%), germacrene D (19.90%) and α-pinene (9.80%)	[50]
*O. caudata* (branches)	Lauraceae	HD	δ-cadinene (13.8%), germacrene D (8.9%), and α-muurulol (7.80%)	[50]
*O. cujumary* (leaves)	Lauraceae	HD	β-caryophyllene (22.20%), caryophyllene oxide (12.40%) and 2-tridecanone (7.30%)	[50]
*O. cujumary* (branches)	Lauraceae	HD	selin-11-en-4-α-ol (20.60%), β-selinene (12.10%) and 7-epi-α-selinene (9.00%).	[50]
*Onychopetalum amazonicum* (leaves)	Annonaceae	HD	(*E*)-caryophyllene (17.00%), caryophyllene oxide (11.90%) and spathulenol (10.40%)	[51]
*O. amazonicum* (trunk bark)	Annonaceae	HD	α-*epi*-cadinol (14.00–24.10%), allo-aromadendrene (21.20%) and α-gurjunene (10.60–14.90%)	[51]
*Piper aequale* (Aerial parts)	Piperaceae	HD	δ-elemeno (18.92%), β-pineno (15.56%), α-pinene (12.57%), cubebol (7.20%), β-atlantol (5.87%) and bicyclogermacrene (5.51%)	[52]
*P. aduncum* (Aerial parts)	Piperaceae	HD	dilapiole (64.40%), piperitone (3.30%) and (*E*)-β-ocimene (3.00%)	[53]
*P. aduncum* (Dry leaves)	Piperaceae	MAE	dilapiol (91.07%)	[54]
*P. aduncum* (Dry leaves)	Piperaceae	SD	dilapiole (53.60%), myristicin (24.30%) and (Z)-carpacin (11.90%)	[55]
*P. aleyreanum* (Aerial parts)	Piperaceae	HD	β-elemene (16.30%), bicyclogermacrene (9.20%), δ-elemene (8.20%), germacrene D (6.90%) and (*E*)-caryophyllene (6.20%)	[12]
*P. anonifolium* (Aerial parts)	Piperaceae	HD	selin-11-en-4-ol (20.00%), β-selinene (12.70%), α-selinene (11.90%) and α-pinene (8.80%).	[12]
*P. augustum* (Leaves)	Piperaceae	HD	(*E)*-caryophyllene (27.10%), germacrene D (11.20%) and β-elemene (5.80%)	[37]
*P. brachypetiolatum* (Fresh Leaves)	Piperaceae	HD	(*E*)-nerolidol (44.23 ± 2.23%) and caryophyllene oxide (10.08 ± 0.74%)	[56]
*P. callosum* (Aerial parts)	Piperaceae	HD	Safrole (69.20%), methyleugenol (8.60%) and myrcene (6.20%)	[53]
*P. capitarianum* (Leaves, stems, and inflorescences)	Piperaceae	HD	(*E*)-caryophyllene (15.30–20.00%), α-humulene (9.10–12.70%), β-myrcene (1.40–10.50%), α-selinene (5.30–7.00%) and β-selinene (4.90–6.30%)	[57]
*P. demeraranum* (dry leaves)	Piperaceae	HD	β-elemene (33.10%), Limonene (19.30%) and bicyclogermacrene (8.80%)	[58]
*P. divaricatum* (Aerial parts)	Piperaceae	HD	methyleugenol (69.20%), eugenol (16.20%) and germacreno D (3.50%)	[53]
*P. duckei* (dry leaves)	Piperaceae	HD	(*E*)-caryophyllene (27.10%), germacrene D (14.70%) and eucalyptol (5.80%)	[58]
*P. glandulosissimum* (Fresh Leaves)	Piperaceae	HD	(*E*)-caryophyllene (19.11 ± 0.40%), α-selinene (8.38 ± 0.17%) and β-selinene (6.38 ± 0.13%)	[56]
*P. hispidum* (Aerial parts)	Piperaceae	HD	(*E*)-caryophyllene (10.50%), α-humulene (9.50%), δ-3-carene (9.10%), α-copaene (7.30%), limonene (6.90%), caryophyllene oxide (5.90%) and β-selinene (5.10%).	[12]
*P. leticianum* (Leaves)	Piperaceae	HD	(*E*)-caryophyllene (21.80%), germacrene D (9.00%) and β-elemene (5.10%)	[37]
*P. madeiranum* (Fresh Leaves)	Piperaceae	HD	caryophyllene oxide (16.92 ± 0.21%), selin-11-en-4-a-ol (9.26 ± 0.12%), β-copaene (9.16 ± 0.12%) and β-selinene (8.70 ± 0.11%).	[56]
*P. marginatum* (Aerial parts)	Piperaceae	HD	*p*-mentha-1(7),8-diene (39.00%) and 3,4-methylenedioxy propiophenone (19.00%),	[53]
*P. marginatum* (Aerial parts)	Piperaceae	HD	(*E*)-isoosmorhizole (32.20%) and (*E*)-anethole (26.40%)	[53]
*P. mollipilosum* (Fresh Leaves)	Piperaceae	HD	β-selinene (32.44 ± 1.14%) and caryophyllene oxide (11.70 ± 0.42%),	[56]
*Psidium guajava*	Myrtaceae	HD	*epi*-β-bisabolol (16.10%), *ar*-curcumene (9.80%), β-bisabolene (9.20%), (*E*)-caryophyllene (5.10%), and caryophyllene oxide (4.50%)	[32]
*P. guineense* Leaves)	Myrtaceae	HD	limonene (30.20–30.4%) and α-pinene (17.70–22.50%)	[32]
*P. myrsinites* (dry Leaves)	Myrtaceae	HD	(*E*)-caryophyllene (26.05%), α-humulene (23.92%) and caryophyllene oxide (10.09%)	[33]
*Renealmia breviscapa* (Fresh rhizomes)	Zingiberaceae	HD	(*E*)-caryophyllene (62.38%), α-Humulene (9.56%) and guaiol (9.27%)	[59]
*R. breviscapa* (fresh leaves)	Zingiberaceae	HD	(*E*)-caryophyllene (28.25%), cis-3-hexenol (15.05%) and bicyclogermacrene (6.90%)	[59]
*R. chrysotricha* (Fresh rhizomes)	Zingiberaceae	HD	α-terpineol (26.14%), coronarin E (25.10%) and eucalyptol (15.87%)	[59]
*R. chrysotricha* (Fresh leavess)	Zingiberaceae	HD	*cis*-3-hexenol (57.28%), (*E*)- caryophyllene (6.85%) and caryophyllene oxide (4.92%)	[59]
*R. nicolaioides* (Fresh rhizomes)	Zingiberaceae	HD	(*E*)-caryophyllene (22.78%), α-terpineol (14.15%) and (*E*)-nerolidol (11.06%)	[59]
*R. nicolaioides* (fresh leaves)	Zingiberaceae	HD	(*E*)-nerolidol (21.03%), α-terpineol (11.92%) and germacrene D (10.33%)	[59]
*Siparuna aspera* (Leaves)	Siparunaceae	HD	germacrene D (23.30%), bicyclogermacrene (7.80%) and α-pinene (7.00%).	[37]
*S. camporum* (dry leaves)	Siparunaceae	HD	γ-patchoulene (28.63%), α-Phellandrene (12.80%) and Guaiadiene-6,9 (9.23%),	[33]
*S. macrotepala* (Leaves)	Siparunaceae	HD	germacrene D (42.10%), bicyclogermacrene (11.80%) and δ-cadinene (5.00%)	[37]
*Syzygium cumini* (leaves)	Myrtaceae	HD	α-pinene	[60]
*Virola calophyla* (leaves)	Myristicaceae	HD	(*E*)-caryophyllene (55.70%) and caryophyllene oxide (9.80%)	[61]
*V. multinervia* (leaves)	Myristicaceae	HD	(*E*)-caryophyllene (54.80%) and bicyclogermacrene (10.00%)	[61]
*V. pavonis* (leaves)	Myristicaceae	HD	β-selinene (60.50%) and (*E*)-caryophyllene (12.70%)	[61]
*V. surinamensis* (barks)	Myristicaceae	HD	Aristolene (28.40 ± 5.03%), α-gurjunene (15.00 ± 3.17%) and valencene (14.10 ± 4.87%).	[62]
*V. surinamensis* (leaves)	Myristicaceae	HD	α-farnensene (14.50 ± 3.24), β-elemene (9.61 ± 1.02%) and bicyclogermacrene (8.10 ± 2.42%).	[62]
*Vismia cayennensis* (Leaves)	Hypericaceae	HD	germacrone (25.42%) and curzerene (25.29%)	[63]
*V. guianensis* (Leaves)	Hypericaceae	HD	α-copaene (29.45%), (*E*)-nerolidol (24.06%) and (*E*)-caryophyllene (10.04%)	[63]
*Xylopia aromatica* (leaves)	Annonaceae	HD	spathulenol (21.50%, *trans*-pinocarveol (10.20%) and dihidrocarveol (11.60%)	[23]

HD: Hydrodistillation; SD: steam distillation; MAE: microwave-assisted extraction.

In the documented studies, the essential oils were obtained by hydrodistillation, except in the case of the species *Copaifera multijuga* (perforation), *Piper aduncum* (MAE), *P. aduncum* (SD), *Ipomea setifera* (SD), and *I. asarifolia* (SD). Gas chromatography coupled with mass spectrometry (GC-MS) was used to identify the volatile compounds in the essential oils. There was little difference in the chemical composition and chemical profile of the essential oils of the species studied based on the families/genera/species, which may be related to the type of botanical material used from the plant in the extraction of the essential oils.

The chemical profile of essential oils from species of the Annonaceae family showed hydrocarbon and oxygenated sesquiterpenes as the main constituents, where the compounds β-bisabolene (55.77%), caryophyllene oxide (55.70%), and β-eudesmol (51.92%), were respectively dominant in the essential oils of *Bocageopsis pleiosperma* [22], *Guatteria blepharophylla* [23], and *G. friesiana* [35]. However, it was possible to observe other types of chemical classes in the genus Anonnace-ae, such as the oxygenated monoterpene *cis*-linalool oxide (33.10%) in the essential oil of *Bocageopsis multiflora* [24] and the alcohol 4-heptanol (33.80%) in the essential oil of *Duguetia quitarensis* [24].

Oxygenated monoterpenes, hydrocarbon sesquiterpenes, and phenylpropanoids are the major components in the essential oils of the Lauraceae family, where linalool (93.60%) is dominant in the essential oil of *Aniba rosaeodora* [16], as well as bicyclogermacrene (42.20%) and apiole (28.10%), respectively, in the essential oil of *Endlicheria arenosa* [28] and *Nectandra puberula* [47]. Phenylpropanoids and oxygenated monoterpenes are also present in essential oils of the Lamiaceae family, where methyleugenol (80.00–87.00%) [48] and eucalyptol (16–33%) are dominant [64].

Studies carried out by Aranha et al. [29] and Da Silva et al. [30] confirmed the predominance of oxygenated sesquiterpenes and hydrocarbons in species of the genus *Eugenia* of the Myrtaceae family. Hydrocarbon sesquiterpenes were also observed as the main chemical classes in the essential oils of the genus *Myrcia*, where (*E*)-caryophyllene (45.80%) was dominant in the essential oil of *M. splendens* [32]. Monoterpene hydrocarbons characterize the essential oil profile of some species of the genus *Psidium* [32].

In species of the Piperaceae family, phenylpropanoids are present in the essential oils of some species of the genus *Piper*, as shown in the study of *Piper aduncum* essential oil by Nascimento et al. [54], the main component of which is dilapiol (91.07%). In species of the family Verbenaceae, the presence of oxygenated monoterpenes such as thymol (63.59–66.20%) was documented in *Lippia thymoides* essential oil [43]. In the species of Zingiberaceae, Siparunaceae, and Myristicaceae, sesquiterpenes are one of the main chemical classes in the chemical profile of the essential oil of some species, especially the compounds (*E*)-caryophyllene (62.38%) [59], and β-selinene (60.50%) [61].

## 3. Antioxidant Activity of Essential Oils

Essential oils comprise different organic compounds that have conjugated carbon double bonds, where the functional species are hydroxyl radicals, which can transfer hydrogen, inhibit free radicals, and minimize oxidative stress [65]. Essential oils with antioxidant properties are preferred over synthetic antioxidants because the former are safer for human health and are eco-friendly [66,67].

Aromatic plants are a well-known source of essential oils with antioxidant properties. These properties are exhibited by the raw essential oils and the isolated chemical constituents, both of which are efficient in preventing lipid oxidation [68]. The antioxidant potential of essential oils can be attributed to a single volatile constituent present in the chemical composition or to the synergistic effect among many components [69]. Table 2 summarizes the antioxidant potential of essential oils from Amazonian plants.

Studies on the antioxidant capacity of essential oils from the Amazon region have shown promising results. da Silva et al. [18] studied the essential oil from both the leaves and branches of *Aniba parviflora*, which strongly inhibited 2,2′-azino-bis(3-ethylbenzothiazoline-6-sulfonic acid) (ABTS) free radicals. The authors indicated that the antioxidant activity may be related to the presence of β-phellandrene, linalool, β-caryophyllene, and γ-eudesmol, which presented antioxidant potential in other documented studies.

The antioxidant potential of some essential oils is equivalent to the inhibition potential of the Trolox standard determined by the 2,2-diphenyl-1-picrylhydrazyl (DPPH) method, as observed for the essential oils of leaves and twigs of *Endlicheria arenosa* [28]. These results may be related to the difference in the chemical composition of the two oils because the chemical profile of the product distilled from the leaves was characterized by the sesquiterpene hydrocarbons bicyclogermacrene (42.2%), germacrene D (12.5%), and β-caryophyllene (10.1%).

Other studies have shown that the inhibition potential of essential oils for the free radicals DPPH and ABTS is higher than that of the Trolox standard, as in the case of the essential oils of *Eugenia patrisii*, *E. punicifolia*, and *Myrcia tomentosa* [31]. Some studies have also reported that a high thymol content may favor higher potential inhibition for essential oils, in which thymol is a major constituent [42]. This is a result of the presence of hydroxyl radicals that facilitate the capture of free radicals and reduce the effects of lipid oxidation [70].

## 4. Biological Activities of Essential Oils from the Amazon Region

### 4.1. Antibacterial Activity

There has been an increasing search for bioactive compounds of natural origin with antimicrobial activities. Natural products and their derivatives are invaluable sources of therapeutic agents [71,72]. In the last few years, essential oils have attracted the interest of researchers because they are composed of mixtures of volatile constituents with potent biological properties, including antibacterial properties [73,74]. The Amazon flora contains several species that are a source of essential oils, some of which have been investigated for their antibacterial activity, as shown in Table 3.

*Ocotea* is a genus of the Lauraceae family that is very important for the economy of the Amazon region. The activity of the essential oils of the leaves of *Ocotea caniculata*, *O. caudalata*, and *O. cujumary* against *Bacillus cereus*, *Escherichia coli*, *Pseudomonas aeruginosa*, *Staphylococcus aureus*, and *Staphylococcus epidermidis* was assessed. The respective oils presented high antimicrobial activity against *Escherichia coli*, with MIC values equal to 19.5 μg/mL for the three species. On the other hand, the essential oil of *Ocotea cujumary* presented moderate activity against *Staphylococcus epidermidis* (MIC = 312.5 μg/mL) and *Bacillus cereus* (MIC = 312.5 μg/mL), and the oil of *O. caudalata* presented moderate activity against *Staphylococcus epidermidis* (MIC = 312.5 μg/mL) [50].

The essential oil of the leaves of *Endlicheria arenosa* (Lauraceae) showed strong antibacterial activity against *Escherichia coli* (MIC = 19.5 μg/mL), and the oils of the leaves and branches showed moderate activity against *Bacillus cereus*, with MIC values of 156 μg/mL for both oils. Other species of the Lauraceae family have also been reported to have antibacterial activity, including *Aniba parviflora, A. rosaeodora*, *Nectandra cuspidata*, and *N. puberula* [17].

Terpenes are the main class of compounds in the essential oils of *Myrcia* (Myrtaceae), and are described in the literature as having inherent antimicrobial properties, as well as synergic action against pathogens in humans. Leomara et al. [10] showed that *Myrcia sylvaltica* essential oils are strong candidates for use individually or in combination with traditional antibiotic products for the manufacture of pharmaceutical products to control strains of resistant bacteria and prevent food deterioration [10].

The essential oil of the fresh and dried leaves of *M. sylvatica* is rich in sesquiterpene hydrocarbons and oxygenated sesquiterpenes, exhibiting activity against *Bacillus cereus* (MIC = 0.2 μL/mL) and *Staphylococcus aureus* (MIC = 2.5 μL/mL) and bacteriostatic potential against *Staphylococcus epidermidis* (20.0 μL/mL) and *Enterococcus faecalis* (20.0 μL/mL) [10]. The essential oil of *M. splendens* also presented a predominance of sesquiterpene compounds, but did not show antibacterial activity against human pathogens; however, it showed moderate activity against phytopathogenic strains such as *Pseudomonas syringae pv. Syringae* (MIC = 250 μg/mL) and *Clavibacter michiganensis subsp. Nebraskensis* (MIC, 125 μg/mL). This activity is related to the major constituent of the oil, *trans*-nerolidol [46].

Bay et al. [24] assessed the antibacterial activity of the essential oils of four species of Annonaceae against *Escherichia coli*, *Pseudomonas aeruginosa*, *Streptococcus mutans*, *Streptococcus pyogenes*, and MRSA. The oil of *Bocageopsis multiflora* was strongly active against the four microorganisms tested. *Duguetia quitarensis* and *Guatteria punctata* were active only against *Streptococcus mutans* and *Streptococcus pyogenes*. The oil of *Fusaea longifolia* showed potential against *Pseudomonas aeruginosa*, *Streptococcus mutans*, and MRSA [24].

Piperaceae is a typical family from tropical regions such as the Amazon. A few studies have pointed out the antimicrobial properties of some species of this family such as the genus *Piper* [75,76].

### 4.2. Antifungal Activity

The use of synthetic fungicides is common on plantations, where this continued use can lead to the development of resistance in fungi, in addition to harming the soil and environment, causing degradation of the medium into which it is discharged [77]. Fungi not only negatively affect plants, but are also harmful to human beings and can cause series of discomfort for their host [78]. For this reason, the bioactivity of essential oils has been increasingly researched, as these oils have promising activity against the action of fungal pathogens, and represent a non-degrading alternative to the environment in the fight against the damage caused by these agents [79]. The antifungal activity of essential oils plausibly results from penetration of chitin in the hyphal wall, triggering a series of damages to the fungal outer wall and destroying it [80].

The essential oils of the aerial parts of Piper divaricatum showed high inhibitory activity against the fungal species Fusarium solani [81]. In another study, the essential oil of P. divaricatum leaves demonstrated significant inhibition of the fungicidal activity of the pathogens Cladosporium cladosporioides and Cladosporium sphareospermum [82]. The antifungal activities of some essential oils from the Amazon are summarized in Table 4.

### 4.3. Cytoxicity

The search for new phytotherapeutics with anticancer (tumor) potential is extremely important because most anticancer drugs are of natural origin. Natural products have a high level of efficacy in use and application, constituting the main ally in the preparation and development of new treatments for cancer [86,87]. In this industry, the essential oils from botanical species of the Amazon region have shown favorable cytotoxic activity and applications, as reported in prior studies [38,88,89], in which the essential oils of two species of *Eugenia* (*E. cuspidifolia* and *E. tapacumensis*) collected in the forest reserve Adolfo Ducke, Manaus, Amazonas, Brazil, were assessed against five types of cancer cells: human malignant melanoma (SK-MEL-19), human colorectal carcinoma (HCT116), human breast adenocarcinoma (MCF7), human gastric adenocarcinoma (ACP02), and human embryonic lung (MRC-5 as a non-malignant cell line). The inhibitory activity of the essential oil of *E. cuspidifolia* (EO1) was demonstrated by the *IC*_50_ values of 18.11 μg mL^−1^ (MCF7), 15.25 μg mL^−1^ (HCT116), 26.17 μg mL^−1^ (SK-MEL-19), >50 μg mL^−1^ (ACP02), and 25.51 μg mL^−1^ (MRC-5). On the other hand, the essential oil of *E. tapacumensis* (EO2) presented inhibitory potential, with *IC*_50_ values of 24.35 μg mL^−1^ (MCF7), 12.37 μg mL^−1^ (HCT116), >50 μg mL^−1^ (SK-MEL-19), >50 μg mL^−1^ (ACP02), and 36.12 μg mL^−1^ (MRC-5). Such results show that EO1 and EO2 from the leaves reduced the viability of HCT116 cells, with *IC_50_* values of 15.25 μg mL^−1^ and 12.37 μg mL^−1^, respectively.

Essential oils from the leaves of *Eugenia patrisii*, *Eugenia stipitata*, *Myrcia splendens*, *Myrcia sylvatica*, *Psidium guajava*, and *Psidium guineense* (Pgui-1 and Pgui-2) were collected from several locations in the cities of Belém/Para/Brazil and Curuçá/Para/Brazil. The activity of the essential oils of these species against five types of cancer cells was analyzed: MCF7 breast cancer, SKMEL-19 melanoma, AGP01Gastric, HCT116 colon cancer, and MRC5 human fibroblasts. The essential oil of *E. patrisii* exhibited no detectable activity against MCF7 breast type cell, but in the other types of cells, it showed the following inhibition potentials: *IC*_50_ = 5.80 μg/mL (SKMEL-19; melanoma), 3.21 μg/mL (AGP01; gastric), 6.70 μg/mL (HCT116; colon), and 3.5 μg/mL (MRC5; human fibroblast). The essential oil of *E. stipitata* did not present cytotoxic activity against AGP01 (gastric) and HCT116 (colon) cells; however, it showed inhibitory activity against the following cells, with *IC*_50_ values of 19.10 μg/mL (MCF7; breast), 17.20 μg/mL (SKMEL-19; melanoma), and 13.8 μg/mL (MRC5; human fibroblast). The essential oil of *M. splendens* exhibited no cytotoxic activity against the MCF7 breast type cell, but showed an inhibition potential of 8.50 μg/mL against (SKMEL-19; melanoma), with *IC*_50_ values of 4.70 μg/mL (AGP01; gastric), 8.80 μg/mL (HCT116; colon), and 6.5 μg/mL (MRC5; human fibroblast). The essential oil of *M. sylvatica* exhibited no detectable activity against (HCT116; colon) type cells; however, the essential oil of such species presented inhibition of >25 μg/mL (MCF7; breast), 20.01 μg/mL (SKMEL-19; melanoma), 17.31 μg/mL (AGP01; gastric), and 23.3 μg/mL (MRC5; human fibroblast). The essential oil of *Psidium guajava*, as well as the essential oil of two specimens of *P. guineense* (Pgui-1 and Pgui-2), did not show cytotoxic activity against cancer cells (HCT116; colon). However, the essential oil of *P. guajava* presented the following inhibition potentials: 12.41 μg/mL (MCF7; breast), 15.31 μg/mL (SKMEL-19; melanoma), 16.31 μg/mL (AGP01; gastric), and 20.8 μg/mL (MRC5; human fibroblast). The specimen (Pgui-1) of *P. guineense* presented inhibition potentials of 11.60 μg/mL (MCF7; breast), 11.10 μg/mL (SKMEL-19; melanoma), 8.21 μg/mL (AGP01; gastric), and 8.27 μg/mL (MRC5; human fibroblast). The Pgui-2specimen presented inhibition potentials of: 18.21 μg/mL (MCF7; breast), 19.11 μg/mL (SKMEL-19; melanoma), 15.71 μg/mL (AGP01; gastric), and 24 μg/mL (MRC5; human fibroblast). The greatest cytotoxic activity was observed for the essential oil of *E. patrisii* against (SKMEL-19; melanoma), (AGP01; gastric), and (HCT116; colon), whereas the essential oils of *P. guajava* and *P. guineense*, were more active against breast cancer cells (MCF7, *IC*_50_ 12.4 µg/mL and 11.6 µg/mL, respectively) [32].

The essential oil of four species of *Eugenia* (*E. egensis*, *E. flavescens*, *E. polystachya*, and *E. patrisii*) collected in Marabá-PA were tested against three types of cancer cells: HCT-116 (colon), SKMEL19 (melanoma), and AGP-01 (gastric). The essential oil of *E. egensis* did not present a cytotoxic profile against the three types of cells, with *IC*_50_ > 25 µg/mL. At the same concentration where *IC*_50_ > 25 µg/mL, the essential oil of *E. flavescens*, *E. polystachya*, and *E. patrisii* did not present cytotoxic activity against the two cancer cells: SKMEL19 (melanoma) and AGP-01 (gastric). On the other hand, the essential oils of *E. flavescens*, *E. patrisii*, and *E. polystachya* showed cytotoxic activity, with *IC*_50_ values of 13.9 µg/mL, 16.4 µg/mL, and 10.3 µg/mL, respectively, against HCT-116 (colon). According to the authors, this cytotoxic potential may be related to the presence of the main compound, germacrene D [30].

The essential oil of *Myrcia splendes* from the equatorial Amazon was assessed against A549 (human lung cancer), MCF-7 (human breast adenocarcinoma), and HaCaT (human keratinocytes) cells. All the results showed inhibition of cancer cell growth depending on the dose of α-bisabolol, which was the most active component. At a concentration of 10 µg/mL, α-bisabolol reduced the viability of A549 (human lung cancer), MCF-7 (human breast adenocarcinoma), and HaCaT (human keratinocytes) cells by 70, 10, and 50%, respectively, compared to the negative control. The growth of MCF-7 type cells was more strongly inhibited than that of the HaCaT cells 48 h after treatment with α-bisabolol (*IC*_50_ = 1.24 ± 0.03 µg/mL vs. 10.15 ± 0.35 µg/mL) and essential oil (*IC*_50_ = 5.59 ± 0.13 µg/mL) vs. 21.58 ± 1.26 µg/mL). However, the HaCaT cells were more sensitive than the A549 cell line, with *IC*_50_ values varying from 10.15 ± 0.35 to 27.76 ± 2.76 µg/mL for the former, compared with values of 54.28 ± 2.39 to 100.99 ± 2.32 µg/mL for the latter. Therefore, the assessment of the cytotoxic activity showed promising results regarding the selectivity and efficacy of the essential oil of *M. splendens* against the cell line MCF-7 compared to that against A549 cells [46].

The essential oils from the leaves of five specimens of *Eugenia uniflora* were collected in Belém and Santarém, Pará, Brazil, and tested against HCT-116 (colon), AGP-01 (malignant gastric ascites), SKMEL-19 (melanoma), and MRC-5 (human fibroblast). The essential oil of specimen E1 did not exhibit cytotoxic activity against the four types of cells, whereas samples E3 and E5 presented equal inhibition percentages (*IC*_50_ > 25 μg/mL) against the four cell types. In contrast, the essential oils of the specimens E2 and E4 showed cytotoxic activity against all the HCT-116 cell lines tested (*IC*_50_ E2: 16.26 μg/mL; E4: 9.28 μg/mL), AGP-01, (*IC*_50_ E2:12.60 μg/mL; E4:8.73 μg / mL), SKMEL-19 (*IC*_50_ E2: 12.20 μg/mL; E4: 15.42 μg/mL), and MRC-5 (*IC*_50_ E2: 10,27 μg/mL; E4: 14.95 μg/mL) [90].

The cytotoxic potential of essential oils from the Piperaceae family, especially the genus piper [91], has been documented [12], in which three species of Piper (*P. hispidum*, *P. aleyreanum*, and *P. anonifolium*) collected in the national forest of Carajás, Pará state, Brazil were tested against three cancer cell lines: HCT-116 (colon), SKMEL19 (melanoma), and ACP-03 (gastric). The essential oils of these three species had low inhibitory effects on the growth of the HCT-116 (colon) and ACP-03 (gastric) cell lines (*IC*_50_ > 25 µg/mL). The oils also had *IC*_50_ > 25 µg/mL for the cell line SKMEL19 (melanoma), except for the essential oil of *P. aleyreanum*, which presented high in vitro cytotoxic activity (*IC*_50_ = 7.4 μg/mL).

The essential oils of the family Lauraceae exhibit cytotoxic activity against some types of cell lines, as shown in a previous study [47], where the essential oils were taken from the leaves and branches of *Nectandra puberula* and only the leaves of *N. Cuspidata*. During this research, the cytotoxic activity of the essential oils from the leaf of *N. puberula* and *N. cuspidata* against MCF-7 breast tumor cells was evaluated, where the *IC*_50_ was 64.5 ± 1.6 and 117.1 ± 11.9 μg/mL, respectively.

The Annonaceae family is characterized by a pantropical family of trees, bushes, and climbers, and is found especially in tropical lowlands [92]. The family is characterized by species rich in essential oils with potential in vitro inhibitory activity against cancer cells [36,92]. This biological activity was observed for the essential oil from the leaves of *Anaxagorea brevipes* collected in Manaus, Amazonas, Brazil. The essential oil showed cytotoxic activity against the MCF-7 (breast, TGI = 12.8 μg/mL), NCI-H460 (lung, TGI = 13.0 μg/mL), and PC-3 (prostate, TGI = 9.6 μg/mL) cell lines [15]. Other botanical families have been studied to prove their efficacy against cancer cells, such as the Myristicaceae family, which is recognized as a species that produces essential oils. The species *Iryanthera polyneura* (Myristicaceae) is commonly known as cumala-colorada, and can be found in the Amazon forest [93]. Studies on this species have shown cytotoxic activity [39] for the essential oil from the leaves of three specimens of *Iryanthera polyneura* collected in Amazonas, Brazil, which were tested against human breast (MCF-7) and prostate (PC-3) cells. In that study, thirty-six of the forty essential oils were more active against PC-3 than against MCF-7 cells, where the samples of the set 22EO, 80EO, and 53EO were particularly active, with inhibition values of *IC*_50_ = 14.69 ± 4.33, 13.63 ± 3.23, and 12.48 ± 4.03 μg/mL, respectively. The essential oils of the leaves and bark of *Virola surinamensis*, native to the Amazon, Brazil, were tested against HCT116 (human colon carcinoma), MCF-7 (human breast adenocarcinoma), HL-60 (human promyelocytic leukemia), HepG2 (human hepatocellular carcinoma), B16–F10 (mouse melanoma), and MRC-5 (human pulmonary fibroblasts). The essential oil of the sample barks presented an inhibition percentage of *IC*_50_ = 9.41 μg/mL against the respective cells. The cytotoxic activities of some essential oils from the Amazon are shown in Table 5.

### 4.4. Antiprotozal Activity

Diseases resulting from protozal infection have caused serious problems and have detrimental impacts on human health. Such diseases include leishmaniasis, which is considered one of the most neglected diseases resulting from the parasitic action of protozoans of the genus *Leishmania* [94]. Within this scope of parasitic diseases, *Trypanossoma cruzi* is predominant in the Americas [95].

The treatment of these diseases is based on highly toxic drugs with little efficacy [96], which cause serious side effects in the body [96]. However, some plants are considered potentially rich and promising for the development of drugs that act against leishmaniosis and Chagas disease [94,96]. In this context, it is important to emphasize that essential oils are substances extracted from aromatic plants and have biological potential against parasites [97]. The biological activity of natural products is related to the active chemical compounds in their composition [98].

Within the Amazon region, studies on the action of essential oils against protozoans are still lacking. However, studies have shown that the essential oils from plants of the Amazon have components that are active against leishmaniosis, as described in a study conducted with the essential oil of *Bocageopsis multiflora*, which presented significant activity (*IC*_50_: 14.6 μg/mL) against promastigotes of *Leishmania amazonenses* [25]. The anti-Leishmania potential of the essential oil of *Syzygium cumini* and its major constituent, α-pinene, was tested, where α-pinene presented an inhibitory concentration of *IC*_50_ = 19.7 mg/mL against the promastigotes of *L. amazonenses*, and *IC*_50_ value of 16.1 mg/mL and 15. mg/mL) against axenic and intracellular amastigotes. On the other hand, the essential oil from *S. cumini* presented inhibitory concentrations of *IC*_50_ = 43.9 mg/mL and *IC*_50_ *=* 38.1 mg/mL against axenic and intracellular amastigotes. According to the authors, α-pinene was the most active substance [60].

The activity of essential oils from two species of Annonaceae, *Guatteria friesiana* (EOGF) and *G. pogonopus* (EOGP), against the protozoa causing malaria (*Plasmodium falciparum*) and Chagas disease (*Trypanosoma cruzi*) was tested. EOGF presented an inhibition potential of *IC*_50_ = 0.53 μg/mL against *P. falciparum* and *IC*_50_ = 10.7 μg/mL against *T. cruzi*. EOGP presented respective *IC*_50_ values of 6.8 and 41.3 μg/mL against *P. falciparum* and *T. cruzi*. According to the authors, EOGF and EOGP presented potent antimalarial and trypanocidal activity [35]. The trypanocidal activity was assessed for essential oils of the leaves and rhizomes of a species of Zingiberaceae (*Renealmia chrysotricha*). At a concentration of 25 µg/mL, the essential oil of the rhizome of *R. chrysotricha* reduced the number of parasites by 50 and 61% after 24 and 48 h, respectively. Treatment with 100 µg/mL reduced the population of parasites by 56% after 24 h, with all parasites eliminated within 48 h. The essential oil of the leaves of *R. chrysotricha* reduced the population of parasites by 28–59% at concentrations of 25, 100, 400, and 800 µg/mL after 24 h, and by 2–53% at concentrations of 25, 100, and 400 µg/mL, with total death of the parasites at 800 µg/mL after 48 h [59].

The essential oil from the leaves and thin branches of three samples of *Aniba rosaeodora* (Lauraceae) and its major constituent linalool were tested against intracellular epimastigote and amastigote forms of *T. cruzi*. In the treatment with the essential oil of *A. rosaeodora*, the inhibitory concentration for the epimastigote forms was *IC*_50_ = 150.5 ± 1.08 µg/mL, and *IC*_50_ = 198.6 ± 1.12 µg/mL for linalool. The essential oil and linalool presented respective inhibitory concentrations of *IC*_50_ = 911.6 ± 1.15 and 249.6 ± 1.18 µg/mL for the intracellular amastigote forms. At higher concentrations, the essential oil and linalool both exhibited antitrypanosomal activity against the intracellular amastigote forms [19].

The activity of the essential oil from the leaves of *Ocimum canum* (Lamiaceae) against the intracellular promastigote and amastigote forms of *Leishmania amazonenses* was assessed. In this study, the essential oil presented respective inhibitory concentrations of *IC*_50_ = 17.4 μg/mL and 13.1 μg/mL for the intracellular promastigote and amastigote forms [49]. In another study, the activity of the essential oils of two species of Piperaceae (*Piper duckei* and *P. demeraranum*) and their major compounds (limonene and *E*-caryophyllene) against strains of *L. amazonenses* and *L. guyanensis* was assessed. Both essential oils reduced the growth of the promastigote forms of two species of leishmania, where the essential oils of *P. duckei* and *P. demeraranum* presented respective inhibitory concentrations of *IC*_50_ = 15.2 μg mL^−1^ and *IC*_50_ = 22.7 μg mL^−1^ for the promastigote forms of *L. guyanensis,* whereas for the amastigote forms of *L. amazonenses,* the inhibitory concentrations were *IC*_50_ = 46.0 μg mL^−1^ and *IC*_50_ = 86.0 μg mL^−1^, respectively. For the amastigote forms of *L. guyanensis*, the essential oils presented inhibitory concentrations of *IC*_50_ = 42.4 μg mL^−1^ for *P. duckei* and *IC*_50_ = 78 μg mL^−1^ for *P. demeraranum*. The major compounds limonene and *E*-caryophyllene respectively exhibited inhibitory concentrations of *IC*_50_ = 278 μM (limonene) and *IC*_50_ =96 μM (*E*-caryophyllene) against the promastigote forms of *L. amazonensis*. Thus, the major compounds presented lower inhibition percentages (*IC*_50_) than the essential oils of *Piper* [58].

### 4.5. Larvicidal Activity and Toxicity

Toxicity studies of essential oils aim to discover new natural insecticidal and larvicidal agents that can fight against several vectors of public health concern [99]. It is important to highlight that these studies have increased steadily due to the strong resistance of microbes to synthetic insecticides that can cause serious problems to the environment, with risk of contamination of the air, soil, and water [65,100]. These problems have expanded the search for and development of natural pesticides, especially aromatic plants in the Amazon region, as described in a study performed with the essential oil of the aerial parts of the species *Mesosphaerum suaveolens* collected in three different periods (intermediate rainy, and dry). The activity of the essential oils against *Aedes aegypti* and *Artemia salina* Leach larvae was tested, demonstrating that the essential oil extracted in the dry season showed greater activity (*LC_50_*) against the larvae of *A. aegypti* (90.9 μg/mL), followed by that obtained in the rainy period (108.0 μg/mL), whereas low activity was observed for the oil acquired in the intermediary period (135.2 μg/mL). In relation to the *Artemia salina* Leach, the essential oil presented moderate toxicity (*LC*_50_) 167.1 μg/mL (intermediary period), 202.6 μg/mL (rainy period), and 215.7 μg/mL (dry period) [45].

Some studies with essential oils of the family Piperaceae native to the Amazon region have demonstrated promising larvicidal activity and toxicity of the essential oil of *Piper capitarianum* in the inflorescence vegetative period, which presented larvicidal potential against *Aedes aegypti* and *Aedes albopictus* (*LC*_50_ = 87.6 μg/mL and 76.1 μg/mL). Likewise, the essential oil obtained from the inflorescence was more active against *Artemia salina* Leach, with an *LC*_50_ of 465.30 μg/mL [57]. In another study, the activity of the essential oils of five species of *Piper* (*P. aduncum*, *P. gaudichaudianum*, *P. malacophyllum*, *P. marginatum*, and *P. tuberculatum*) against one type of rice blight (*Tibraca limbativentris*) was tested. The essential oils significantly reduced the hatching of *T. limbativentris* eggs, with *LC*_50_ = 2.49 μg/mL (*P. aduncum),* 4.243 μg/mL (*P. gaudichaudianum*), 6.073 μg/mL (*P. malacophyllum*), 1.968 μg/mL (*P. marginatum*), and 3.388 μg/mL (*P. tuberculatum)*. The results demonstrate that essential oils are promising for use as botanical insecticides [101]. The essential oil of *Piper aduncum* presented insecticidal potential against one type of soybean pest, *Chrysodeixis includens* Walker, with *LC*_50_ = 3.5 μg/mL. According to the authors, further studies are necessary to confirm the use of this essential oil, rather than synthetic chemical products, to control this pest [55].

The insecticidal activity of the essential oils of Piper (*P. aduncum*, *P. marginatum* (chemotypes A and B), *P*. *divaricatum*, and *P. callosum*) against the termite *Solenopsis saevissima* was assessed. The activity values were *LC*_50_ = 114.4 μg/mL (*P. aduncum*), *LC*_50_ = 207.8 μg/mL (*P. marginatum* A), *LC*_50_ = 419.3 μg/mL (*P. marginatum* B), *LC*_50_ = 552.2 μg/mL (*P*. *divaricatum*), and *LC*_50_ = 571.1 μg/mL (*P. callosum*). The authors suggested new investigations of these essential oils for use in sustainable pest control in the Amazon region [53].

The larvicidal potential of essential oils from the leaves of three specimens of *Virola* (*V. calophylla*, *V. multinervia*, and *V. pavonis*) was tested to verify their activity against *A. aegypti*. The essential oil of *V. calophylla* presented *LC*_50_ = 179.6 μg/mL, followed by that of *V. pavonis LC*_50_ = 185.1 μg/mL and *V. multinervia LC*_50_ = 200.5 μg/mL. According to the authors, the essential oil of *Virola* had low larvicidal potential [61]. In contrast, the essential oil of *Bauhinia ungulata* (Fabaceae) presented high toxicity against *Artemia salina* Leach, with *LC*_50_ = 144.75 µg mL^−1^ [21].

Dias et al. [33] assessed the insecticidal potential of essential oils of *Eugenia piauhiensis*, *Myrcia erythroxylon*, *Psidium myrsinites*, *Siparuna camporum*, and *Lippia gracilis* against larvae of *A. aegypti* [33]. The essential oil of *M. erythroxylon* was inactive against *A. aegypti* larvae, with *LC*_50_ > 1000 mg/L, whereas the other essential oils were considered effective, with *LC*_50_ = 230, 251, 282, and 292 mg/L, respectively, for *E. piauhiensis*, *S. camporum*, *L. gracilis*, and *P. myrsinites.* The essential oil of the leaves and branches of *Aniba duckei* showed larvicidal activity against *A. aegypti*, with *LC*_50_ = 250.6 µg mL^−1^ [16]. Likewise, the essential oil of *Lippia origanoides* presented larvicidal potential against *Cerataphis lataniae* within 24 h of exposure, with *LD*_50_ = 6.6 μg/mL and *LD*_90_ = 41.9 μg/mL, and *LD*_50_ = 2.7 μg/mL and *LD*_90_ = 19.8 μg/mL within 48 h of exposure [41].

## 5. Conclusions

The Amazon flora has a wide range of aromatic plants with potential application in the international and national markets due to their fragrances and aromas and for their use in the traditional medicine for the treatment of several diseases. The essential oils and their compounds are directly related to the bioactive compounds found in the essential oils of the Amazon biome. The chemical profile of the essential oils extracted from amazon species is characterized specially by the terpenes, monoterpenes, sesquiterpenes, and phenylpropanoids. Therefore, the essential oils listed in the present study show a great potential for the development of natural pesticides, antioxidant products, and drugs with antimicrobial and cytotoxic effect.

## Figures and Tables

**Table 2:** Essential oils of the Amazon and their antioxidant activities.

Species (Plants Part)	Family	Method	Results	References
*Aniba parviflora* (Leaves)	Lauraceae	DPPH	TEAC = 90.1–287.9 mg TE/mL	[18]
*A. parviflora* (Branches)	Lauraceae	DPPH	TEAC = 94.1–358.4 mg TE/mL	[18]
*A. rosaeodora* (Aerial parts)	Lauraceae	ABTS	EC_50_ = 15.46 µg/mL	[19]
*Endlicheria arenosa* (Leaves)	Lauraceae	DPPH	TEAC = 334.1 ± 41.6 mg TE/mL	[28]
*E. arenosa* (Twigs)	Lauraceae	DPPH	TEAC = 252.6 ± 24.4 mg TEmL	[28]
*Eugenia egensis* (Aerial parts)	Myrtaceae	DPPH	TEAC = 216.5 ± 11.6 mg TE/mL	[30]
*E. flavescens* (Aerial parts)	Myrtaceae	DPPH	TEAC = 122.6 ± 6.8 mg TE/mL	[30]
*E. patrisii* (Aerial parts)	Myrtaceae	DPPH	TEAC = 111.2 ± 12.4 mg TE/mL	[30]
*E. patrisii* (Leaves)	Myrtaceae	DPPH	Inhibition = 28.9 ± 4.8%	[32]
*E. patrisii* (Dry leaves)	Myrtaceae	DPPH	Inhibition = 99.0 ± 0.099% (Specimen A) Inhibition = 204.0 ± 0.877% (Specimen B)	[31]
		ABTS	Inhibition = 31.4 ± 0.1% (Specimen A) Inhibition = 17.9 ± 0.069% (Specimen B)	
*E. punicifolia* (Dry leaves)	Myrtaceae	DPPH	Inhibition = 408.0 ± 0.10% (Specimen A) Inhibition = 285.0 ± 0.028% (Specimen B)	[31]
		ABTS	Inhibition = 9.5 ± 0.034% (Specimen A) Inhibition = 37.7 ± 0.035% (Specimen B)	
*E. uniflora* (Leaves)	Myrtaceae	DPPH	Inhibition = 42.6 ± 0.3 to 64.2 ± 0.3%	[34]
*E. uniflora* (Dry leaves)	Myrtaceae	DPPH	Inhibition = 30.3 ± 3.3 to 40.6 ± 1.9%	[48]
		β-Carotene	Inhibition = 153.5 ± 16.5 to 228.3 ± 19.2%	
		MTT	Inhibition = 10.8 ± 3.4 to 26.3 ± 1.2%	
*Hedychium coronarium* (Rhizome)	Zingiberaceae	DPPH	IC_50_ = 9.04 ± 0.55 mg/mL	[37]
		ABTS	IC_50_ = 2.87 ± 0.17 mg/mL	
*Lippia thymoides* (Fresh Leaves)	Verbenaceae	DPPH	Inhibition = 89.97 ± 0.31%	[42]
*L. thymoides* (Dry leaves)	Verbenaceae	DPPH	Inhibition = 63.53 b ± 5.04–73.63 ± 2.09%	[42]
*Mentha piperita* (Dry leaves)	Lamiaceae	DPPH	AA = 79.9 ± 1.6%	[44]
*Myrcia splendens* (Leaves)	Myrtaceae	DPPH	Inhibition = 28.4 ± 7.1%	[32]
*M. sylvatica* (Leaves)	Myrtaceae	DPPH	Inhibition = 18.5 ± 3.5%	[32]
*M. tomentosa* (Dry leaves)	Myrtaceae	DPPH	Inhibition = 213.0 ± 0.905% (Specimen A) Inhibition = 208.5 ± 0.940% (Specimen B)	[31]
		ABTS	Inhibition = 53.6 ± 0.150% (Specimen A) Inhibition = 0.333 ± 0.247% (Specimen B)	
*Ocimum campechianum* (leaves and stems and inflorescences)	Lamiaceae	DPPH	Inhibition = 36.0% (leaves and stems) Inhibition = 41.6% (inflorescences)	[48]
			TEAC = 58.5 mgTE/mL (leaves and stems) TEAC = 68.4 mgTE/mL (inflorescences)	
*Piper aequale* (Aerial parts)	Piperaceae	DPPH	TEAC = 280.9 ± 22.2 mg TE/mL	[52]
*P. aleyreanum* (Aerial parts)	Piperaceae	DPPH	TEAC = 412.2 ± 9.5 mg TE/mL	[12]
*P. anonifolium* (Aerial parts)	Piperaceae	DPPH	TEAC = 148.6 ± 26.9 mg TE/mL	[12]
*P. augustum* (Leaves)	Piperaceae	DPPH	IC_50_ = 6.17 ± 0.33 mg/mL	[37]
		ABTS	IC_50_ = 2.16 ± 0.20 mg/mL	
*P. brachypetiolatum* (Fresh Leaves)	Piperaceae	DPPH	EC_50_ = 64.8 ± 3.8 µg/mL	[56]
		ABTS	EC_50_ = 159.7 ± 8.3 µg/mL	
*P. glandulosissimum* (Fresh Leaves)	Piperaceae	DPPH	EC_50_ = 104.4 ± 6.4 µg/mL	[56]
		ABTS	EC_50_ = 200.9 ± 6.4 µg/mL	
*P. hispidum* (Aerial parts)	Piperaceae	DPPH	TEAC = 303.1 ± 49.2 mg TE/mL	[12]
*P. leticianum* (Leaves)	Piperaceae	DPPH	IC_50_ = 4.26 ± 0.11 mg/mL	[37]
		ABTS	IC_50_ = 2.65 ± 0.25 mg/mL	
*P. madeiranum* (Fresh Leaves)	Piperaceae	DPPH	EC_50_ = 66.8 ± 5.2 µg/mL	[56]
		ABTS	EC_50_ = 242.6 ± 6.8 µg/mL	
*P. mollipilosum* (Fresh Leaves)	Piperaceae	DPPH	EC_50_ = 79.0 ± 4.9 µg/mL	[56]
		ABTS	EC_50_ = 280.5 ± 6.6 µg/mL	
*Psidium guajava* (Leaves)	Myrtaceae	DPPH	Inhibition = 38.6 ± 7.0%	[32]
*P. guineense*	Myrtaceae	DPPH	Inhibition = 11.5 ± 2.0% (Pgui-1) Inhibition = 27.7 ± 2.3% (Pgui-2)	[32]
*Siparuna aspera* (Leaves)	Siparunaceae	DPPH	IC_50_ = 20.70 ± 0.80 mg/mL	[37]
		ABTS	IC_50_ = 1.12 ± 0.04 mg/mL	
*S. macrotepala* (Leaves)	Siparunaceae	DPPH	IC_50_ = 29.37 ± 1.15 mg/mL	[37]
		ABTS	IC_50_ = 0.80 ± 0.03 mg/mL	

DPPH, 2,2-Diphenyl-1-picrylhydrazyl; ABTS, 2,2-azinobis-(3-ethylbenzothiazoline-6-sulfonate); *EC*_50_ (concentration required to obtain 50% antioxidant effect).

**Table 3:** Antibacterial activity of essential oils from species found in the Amazon.

Species	Family	Methodos	Microrganisms (Results)	References
*Anaxagorea brevipes*	Annonaceae	Microbroth dilution	*Kocuria rhizophila* (MIC = 50.00 μg/mL)	[15]
(Leaves)			*Staphylococcus aureus* (MIC = 250.00 μg/mL)	
			*Staphylococcus aureus penicillinase-negative* (8−) (MIC = 25.00 μg/mL)	
			*Staphylococcus aureus penicillinase-positive* (7+) (MIC = 250.00 μg/mL)	
			*Enterococcus faecalis* (MIC = 250.00 μg/mL)	
*Aniba parviflora*	Lauraceae	Agar disk diffusion/The plate	*Klebsiella pneumoniae* (DDM = 9.20 mm/MIC = >10 μL/mL)	[17]
(Aerial parts)		microdilution	*Staphylococcus aureus* (DDM = 15.44 mm/MIC = >10 μL/mL)	
			*Enterococcus faecalis* (DDM = 11.2 mm/MIC = >10 μL/mL)	
			*Staphylococcus epidermidis* (DDM = 13.3 mm/MIC = >10 μL/mL)	
			*Streptococcus pyogenes* (DDM = 13.3 mm/MIC = 1.3 μL/mL)	
*A. rosaeodora*	Lauraceae	Agar disk diffusion	*Escherichia coli* (DDM =13.2 mm/MIC = >10 μL/mL)	[17]
(Aerial parts)			*Klebsiella pneumoniae* (DDM = 11.6 mm/MIC = > 10 μL/mL)	
			*Staphylococcus aureus* (DDM = 26.7 mm/MIC = 1.3 μL/mL)	
			*Enterococcus faecalis* (DDM = 8.80 mm/MIC = 5 μL/mL)	
			*Staphylococcus epidermidis* (DDM = 38.4 mm/MIC = 5 μL/mL)	
			*Streptococcus pyogenes* (DDM = >40/MIC = 1.3 μL/mL)	
*Bocageopsis pleiosperma*	Annonaceae	Microbroth dilution	*Staphylococcus epidermidis* (MIC = 250 μg/mL)	[22]
(Barks)				
*B. multiflora*	Annonaceae	Microdilution	*Staphylococcus aureus* (MIC = 0.19 mg/mL)	[23]
(Leaves)			*Enterococcus faecalis* (MIC = 0.09 mg/mL)	
			*Streptococcus sanguinis* (MIC = 0.19 mg/mL)	
			*Pseudomonas aeruginosa* (MIC = 3.0 mg/mL)	
			*Escherichia coli* (MIC = 1.5 mg/mL)	
			*Salmonella enterica* (MIC = 1.5 mg/mL)	
*B. multiflora*	Annonaceae	Microdilution	*Escherichia coli* (MIC = 4.68 μg/mL)	[24]
(Aerial parts)			*Pseudomonas aeruginosa* (MIC = 4.68 μg/mL)	
			*Streptococcus mutan* (MIC = 4.68 μg/mL)	
			*streptococcus pyogenes* (MIC = 4.68 μg/mL)	
			MRSA (MIC = 4.68 μg/mL)	
*Duguetia quitarensis*	Annonaceae	Microdilution	*Streptococcus mutan* (MIC = 37.5 μg/mL)	[24]
(Aerial parts)			*Streptococcus pyogenes* (MIC = 37.5 μg/mL)	
*Ephedranthus amazonicus*	Annonaceae	Microdilution	*Staphylococcus aureus* (MIC = 0.09 g/mL)	[23]
(Leaves)			*Enterococcus faecalis* (MIC = 0.19 mg/mL)	
			*Streptococcus sanguinis* (MIC = 2.50 mg/mL)	
			*Pseudomonas aeruginosa* (MIC = 3.0 mg/mL)	
			*Escherichia coli* (MIC = 1.5 mg/mL)	
*Endlicheria arenosa*	Lauraceae	Microbroth dilution	*Pseudomonas aeruginosa* (MIC = 1250.0 μg/Ml	[28]
(Leaves)			*Escherichia coli* (MIC =19.5 μg/mL)	
			*Staphylococcus epidermidis* (MIC = 625.0 μg/mL)	
			*Staphylococcus aureus* (MIC = 625.0 μg/mL)	
			*Salmonella enterica* (MIC = 1.5 mg/mL)	
*E. arenosa*	Lauraceae	Microbroth dilution	*Pseudomonas aeruginosa* (MIC = 1250.0 μg/Ml	[28]
(Twigs)			*Staphylococcus aureus* 625.0 μg/mL	
*Fusaea longifolia*	Annonaceae	Microdilution	*Pseudomonas aeruginosa* (MIC = 37.5 μg/mL)	[24]
(Aerial parts)			*Streptococcus mutan* (MIC = 37.5 μg/mL)	
			MRSA (MIC = 37.5 μg/mL)	
*Guatteria blepharophylla*	Annonaceae	Microbroth dilution	*Staphylococcus aureus* (MIC = 0.05 mg/mL)	[23]
(Leaves)			*Enterococcus faecalis* (MIC = 0.05 mg/mL)	
			*Streptococcus sanguinis* (MIC = 0.02 mg/mL)	
			*Pseudomonas aeruginosa* (MIC = 1.5 mg/mL)	
			*Escherichia coli* (MIC = 1.5 mg/mL)	
			*Salmonella enterica* (MIC = 1.5 mg/mL)	
*G. punctata*	Annonaceae	Microdilution	*Streptococcus mutan* (MIC = 4.68 μg/mL)	[24]
(Aerial parts)			*Streptococcus pyogenes* (MIC = 4.68 μg/mL)	
*Lippia origanoides*	Verbenaceae	Microbroth dilution	*Staphylococcus aureus* (MIC = 1.15 mg/mL)	[40]
(Aerial parts)			*Enterococcus faecalis* (MIC = 0.57 mg/Ml)	
			*Escherichia coli* (MIC = 1.15 mg/mL)	
			*Klebsiella pneumoniae* (MIC = 1.15 mg/mL)	
*Myrcia splendens*	Myrtaceae	Microdilution	*Agrobacterium tumefaciens* (MIC = 500 μg/mL)	[46]
(Leaves)			*Agrobacterium vitis* (MIC = 2000 μg/mL)	
			*Pseudomonas syringaepv.syringae* (MIC = 250 μg/mL)	
			*Escherichia coli* (MIC = >2000 μg/mL)	
			*Pseudomonas aeruginosa* (MIC = >2000 μg/mL)	
			*Lavibacter michiganensissubsp.nebraskensis* (MIC = 125 μg/mL)	
			*Enterococcus faecalis* (MIC = 2000 μg/mL)	
			*Listeria grayi* (MIC = 1000 μg/mL)	
			*Staphylococcus aureus* (MIC = 1000 μg/mL)	
			*Staphylococcus epidermidis* (MIC = 1000 μg/mL)	
*Myrcia sylvatica*	Myrtaceae	Disk method	*Staphylococcus aureus* (MIC = 2.5 μL/mL)	[10]
(Fresh leaves)			*Staphylococcus epidermidis* (MIC =20 μL/mL)	
			*Bacillus cereus* (MIC = 0.2 μL/mL)	
			*Enterococcus faecalis* (MIC = 20 μL/mL)	
*M. sylvatica*	Myrtaceae	Disk method	*Staphylococcus aureus* (MIC = 2.5 μL/mL)	[10]
(Dried Leaves)			*Staphylococcus epidermidis* (MIC = 20 μL/mL)	
			*Bacillus cereus* (MIC = 0.2 μL/mL	
			*Enterococcus faecalis* (MIC = 20 μL/mL)	
*Nectandra cuspidata*	Lauraceae	Microbroth dilution	*Pseudomonas aeruginosa* (MIC = 1250.0 μg/mL)	[47]
*(Leaves)*			*Escherichia coli* (MIC= 19.5 μg/mL)	
			*Staphylococcus epidermidis* (MIC = 1250.0 μg/mL)	
			*Staphylococcus aureus* (MIC = 625.0 μg/mL)	
			*Bacillus cereus* (MIC = 312.5 μg/mL)	
*N. puberula*	Lauraceae	Microbroth dilution	*Pseudomonas aeruginosa* (MIC = 1250.0 μg/mL)	[47]
*(Leaves)*			*Escherichia coli* (MIC = 19.5 μg/mL)	
			*Staphylococcus epidermidis* (MIC = 1250.0 μg/mL)	
			*Staphylococcus aureus* (MIC = 625.0 μg/mL)	
			*Bacillus cereus* (MIC = 625.0 μg/mL)	
*Ocotea Caniculata*	Lauraceae	Microbroth dilution	*Pseudomonas aeruginosa* (MIC = 1250.0 μg/mL)	[50]
*(Leaves)*			*Escherichia coli* (MIC = 19.5 μg/mL)	
			*Staphylococcus epidermidis* (MIC = 625.0 μg/mL)	
			*Staphylococcus aureus* (MIC = 625.0 μg/mL)	
			*Bacillus cereus* (MIC = 625.0 μg/mL)	
*O. caudalata*	Lauraceae	Microbroth dilution	*Pseudomonas aeruginosa* (MIC = 1250.0 μg/mL)	[50]
(Leaves)			*Escherichia coli* (MIC = 19.5 μg/mL)	
			*Staphylococcus epidermidis* (MIC = 625.0 μg/mL)	
			*Staphylococcus aureus* (MIC = 625.0 μg/mL)	
			*Bacillus cereus* (MIC = 312.5 μg/mL)	
*O. cujumary*	Lauraceae	Microbroth dilution	*Pseudomonas aeruginosa* (MIC = 1250.0 μg/mL)	[50]
(Leaves)			*Escherichia coli* (MIC = 19.5 μg/mL)	
			*Staphylococcus epidermidis* (MIC = 625.0 μg/mL)	
			*Staphylococcus aureus* (MIC = 625.0 μg/mL)	
			*Bacillus cereus* (MIC = 312.5 μg/mL)	
*Onychopetalum amazonicum*	Annonaceae	Microbroth dilution	*Staphylococcus epidermidis* (MIC = 62.5 μg/mL)	[51]
(trunk bark)			*kocuria rhizophila* (MIC = 62.5 μg/mL)	
			*Escherichia coli* (MIC = 62.5 μg/mL)	
*Vismia cayennensis*	Hypericaceae	Microplate dilution	*Staphylococcus aureus* (MIC = >25 μg/mL)	[63]
(Leaves)			*Escherichia coli* (MIC = >50 μg/mL)	
*V. guianensis*	Hypericaceae	Microplate dilution	*Staphylococcus aureus* (MIC = >1000 μg/mL)	[63]
(Leaves)			*Escherichia coli* (MIC = >1000 μg/mL)	
*Xylopia aromatica*	Annonaceae	Microdilution	*Staphylococcus aureus* (MIC = 1.20 mg/mL)	[63]
(Leaves)			*Enterococcus faecalis* (MIC = 0.05 mg/mL)	
			*Streptococcus sanguinis* (MIC = 0.02 mg/mL)	
			*Pseudomonas aeruginosa* MIC = 3.0 mg/mL)	
			*Escherichia coli* (MIC = 3.0 mg/mL)	
			*Salmonella enterica* (MIC = 1.5 mg/mL)	

MIC, minimum inhibitory concentration; DDM, disk diffusion method.

**Table 4:** Antifungal activity of essential oils from the Amazon.

Species	Family	Methodos	Microrganisms (Results)	References
	Fabaceae	ASD	*Aspergillus flavus* (MIC = 0.08 mg/mL—19.5 ± 2.1)	[26]
*Copaifera multijuga*			*Aspergillus níger* (MIC = 0.1 mg/mL—9.5 ± 0.7)	
(resin)			*Aspergillus tamarii* (MIC = 0.5 mg/mL—9.0 ± 0.0)	
			*Aspergillus tamarii* (MIC = 0.3 mg/mL—12.5 ± 3.5)	
			*Aspergillus terréus* (MIC = 0.3 mg/mL—11.5 ± 2.1)	
			*Candida guilliermondii* (MIC = 0.1 mg/mL—9.5 ± 1.1)	
			*Candida tropicallis* (MIC = 0.5 mg/mL—10.0 ± 0.0)	
			*Candida parapsilosis* (MIC = 0.1 mg/mL—16.0 ± 1.4)	
*Ocimum compechianum* (leaves/stems)	Lamiacea	PDA	Growth (%) *Fusarium oxysporum*	[48]
			(IC50 0.25 µL/mL—23.9 ± 3.8)	
			(IC50 0.50 µL/Ml—47.1 ± 6.2)	
			(IC50 0.75 µL/mL—59.4 ± 1.2)	
			(IC50 1.00 µL/mL—60.8 ± 3.7)	
			(IC50 2.50 µL/mL—70.3 ± 8.7)	
*O. compechianum*	Lamiacea	PDA	Germination (%) *Fusarium oxysporum*	[48]
(leaves/stems)			(IC50 0.50 µL/mL—22.6 ± 1.6)	
			(IC50 0.75 µL/mL—38.1 ± 11.6)	
			(IC50 1.00 µL/mL—33.0 ± 1.7)	
			(IC50 2.50 µL/mL—58.7 ± 0.0)	
*O. compechianum*	Lamiacea	PDA	Growth (%) *Colletotrichum gossypii*	[48]
(leaves/stems)			(IC50 0.25 µL/mL—0.0 ± 0.0)	
			(IC50 0.50 µL/mL—31.5 ± 1.5)	
			(IC50 0.75 µL/mL—50.7 ± 8.7)	
			(IC50 1.00 µL/mL—55.0 ± 3.3)	
			(IC50 2.50 µL/mL—100.0 ± 0.0)	
	Lauraceae	PDA	*Fusarium oxysporum* f. sp. *dianthi*—Innibition: 31.2 ± 0.45%	[83]
*Ocotea longifólia*				
(leaves)			*Botrytis cinerea*em—Innibition: 32.8 ± 0.21%	
*O. macrophylla*	Lauraceae	PDA	*Fusarium oxysporum* f. sp. *dianthi*—Innibition: 13.2 ± 0.32%	[83]
(leaves)			*Botrytis cinerea*em—Innibition: 13.2 ± 0.32%	
*Piper aduncum*	Piperaceae	TLC plates	*Cladosporium cladosporioides* (DL = 100 µg)	[82]
(aerial parts)			*Cladosporium sphareospermum* (DL = 100 µg)	
*P. aleyreanum*	Piperaceae	TLC plates	*Cladosporium cladosporioides* (DL = <0.1)	[12]
(aerial parts)			*Cladosporium sphareospermum* (DL = <0.1)	
*P. divaricatum*	Piperaceae	MIC	(MIC = 0.50 mg/mL = 38.93 ± 4.77)	[81]
(aerial parts)			*F. solani f.* sp. *piperis* (MIC = 0.75 mg/mL = 63.36 ± 0.00)	
			(MIC =1.00 mg/mL = 77.10 ± 10.49)	
			(MIC = 2.50 mg/mL = 92.37 ± 3.50)	
*P. divaricatum*	Piperaceae	TLC plates	*C. cladosporioides* (MIC = 0.5 µg)	[82]
(leaves)			*C. sphaerospermum* (MIC = 5.0 µg)	
*P. hispidum*	Piperaceae	TLC plates	*Cladosporium cladosporioides* (DL = 0.1)	[12]
(aerial parts)			*Cladosporium sphareospermum* (DL = 1.0)	
*P. krukoffii*	Piperaceae	TLC plates	*C. cladosporioides* (MIC = 0.1 μg/mL)	[84]
(twig)			*C. sphaerospermum* (MIC = 0.1 μg/mL)	
*P. krukoffii*	Piperaceae	TLC plates	*C. cladosporioides* (MIC = 0.5 μg/mL)	[84]
(leaves)			*C. sphaerospermum* (MIC = 0.5 μg/mL)	
*P. marginatum*	Piperaceae	TLC plates	*C. cladosporioides* (DL = 10 μg/mL)	[85]
(aerial parts)			*C. sphaerospermum* (DL = 25 μg/mL)	

MIC, minimum inhibitory concentration; DDM, disk diffusion method.

**Table 5:** Cytotoxic activity of essential oils from species found in the Amazon.

Species	Botanic Family	Methodos	Results	References
*Anaxagorea brevipes*	Anonnaceae	SRB assay	MCF-7 = TGI 12.8 μg/mL)	[15]
			NCI-H460 = (TGI 13.0 μg/mL)	
			PC-3 = TGI 9.6 μg/mL)	
*Eugenia cuspidifolia*	Myrtaceae	Alamar blue assay	(MCF7) = *IC*_50_ 18.11 μg mL^−1^	[29]
			(HCT116) = *IC*_50_ 15. 25 μg mL^−1^	
			(SK-MEL-19) = *IC*_50_ 26.17 μg mL^−1^	
			(ACP02) = *IC*_50_ > 50 μg mL^−1^	
			(MRC-5) = *IC*_50_ 25.51 μg mL^−1^	
*E. egensis*	Myrtaceae	MTT colorimetric assay	HCT-116 = *IC*_50_ > 25 µg/mL	[30]
			SKMEL19 = *IC*_50_ > 25 µg/mL	
			AGP-01 = *IC*_50_ > 25 µg/mL	
*E. flavescens*	Myrtaceae		HCT-116 = *IC*_50_ 13.9 µg/mL	[30]
			SKMEL19 = ****	
			AGP-01 = ****	
*E. patrisii*	Myrtaceae		MCF7 = ****	[32]
			SKMEL-19 = *IC*_50_ 5.80 μg/mL	
			AGP01 = *IC*_50_ 3.21 μg/mL	
			HCT116 = *IC*_50_ 6.70 μg/mL	
			MRC5 = *IC*_50_ 3.5 μg/mL	
*E. patrisii*	Myrtaceae		HCT-116 = *IC*_50_ 16.4 µg/mL	[30]
			SKMEL19 = ****	
			AGP-01 = ****	
*E. polystachya*	Myrtaceae		HCT-116 = *IC*_50_ 10.3 µg/mL	[30]
			SKMEL19 = ****	
			AGP-01 = ****	
*E. stipitata*	Myrtaceae		MCF7 = *IC*_50_ 19.10 μg/mL	[32]
			SKMEL-19 = *IC*_50_ 17.20 μg/mL,	
			AGP01 = ****	
			HCT116 = ****	
			MRC5 = I*C*_50_ 13.8 μg/mL	
*E. tapacumensis*	Myrtaceae	Alamar blue assay	(MCF7) = *IC*_50_ 24.35 μg mL^−1^	[29]
			(HCT116) = *IC*_50_ 12.37 μg mL^−1^	
			(SK-MEL-19) = *IC*_50_ > 50 μg mL^−1^	
			(ACP02) *IC*_50_ > 50 μg mL^−1^	
			(MRC-5) = *IC*_50_ 36.12 μg mL^−1^	
*E. uniflora*	Myrtaceae	MTT colorimetric assay	HCT-116 (*IC*_50_ E2: 16.26 μg/mL; *IC*_50_ E4: 9.28 μg/mL)	[90]
			AGP-01, (*IC*_50_ E2: 12.60 μg/mL; *IC*_50_ E4: 8.73 μg/mL)	
			SKMEL-19 (*IC*_50_ E2: 12.20 μg/mL; *IC*_50_ E4: 15.42 μg/mL)	
			MRC-5 (*IC*_50_ E2: 10,27 μg/mL; *IC*50 E4: 14.95 μg/mL)	
*Iryanthera polyneura*	Myristicaceae	SRB assay	PC-3 = *IC*_50_ 14.69 ± 4.33 μg/mL	[39]
			MCF-7 = *IC*_50_ 13.63 ± 3.23 μg/mL	
*Myrcia splendens*	Myrtaceae	MTT colorimetric assay	MCF7 = ****	[32]
			SKMEL-19 = *IC*_50_ 8.50 μg/mL	
			AGP01 = *IC*_50_ 4.70 μg/mL	
			HCT116 = *IC*_50_ 8.80 μg/mL	
			MRC5 = *IC*_50_ 6.5 μg/mL	
*M. splendens*	Myrtaceae		A549 = *IC*_50_ 54.28 ± 2.39 µg/mL	[46]
			MCF-7 = *IC*_50_ 1.24 ± 0.03 µg/mL	
			HaCaT = *IC*_50_ 10.15 ± 0.35 µg/mL	
*M. sylvatica*	Myrtaceae		MCF7 = *IC*_50_ > 25 μg/mL	[32]
			SKMEL-19 = *IC*_50_ 20.01 μg/mL	
			AGP01 = *IC*_50_ 17.31 μg/mL	
			HCT116 = ****	
			MRC5 = *IC*_50_ 23.3 μg/mL	
*Nectandra cuspidata*	Lauraceae		MCF-7 = *IC*_50_ 117.1 ± 11.9 μg mL^−1^	[47]
*N. puberula*			MCF-7 = *IC*_50_ 64.5 ± 1.6 μg mL^−1^	
*Piper anonifolium*	Piperaceae		HCT-116 = *IC*_50_ > 25 µg/mL	[12]
			ACP-03 = *IC*_50_ > 25 µg/mL	
			SKMEL19 = *IC*_50_ > 25 µg/mL	
*P. aleyreanum*			HCT-116 = *IC*_50_ > 25 µg/mL	
			ACP-03 = *IC*_50_ > 25 µg/mL	
			SKMEL19 = *IC*_50_ = 7.4 μg/mL	
*P. hispidum*			HCT-116 = *IC*_50_ > 25 µg/mL	
			ACP-03 = *IC*_50_ > 25 µg/mL	
			SKMEL19 = *IC*_50_ > 25 µg/mL	
*Psidium guajava*	Myrtaceae		MCF7 = *IC*_50_12.41 μg/mL	[32]
			SKMEL-19 = *IC*_50_ 15.31 μg/mL	
			AGP01 = *IC*_50_ 16.31 μg/mL	
			HCT116 = ****	
			MRC5 = *IC*_50_ 20.8 μg/mL	
*P. guineense* (Pgui-1)			MCF7 = *IC*_50_ 11.60 μg/mL	
			SKMEL-19 = *IC*_50_ 11.10 μg/mL	
			AGP01 = *IC*_50_ 8.21 μg/mL	
			HCT116 = ****	
			MRC5 = *IC*_50_ 8.27 μg/mL	
*P. guineense* (Pgui-2)			MCF7 = *IC*_50_ 18.21 μg/mL	
			SKMEL-19 = *IC*_50_ 19.11 μg/mL	
			AGP01 = *IC*_50_ 15.71 μg/mL	
			HCT116 = ****	
			MRC5 = 24 μg/mL	
*Virola surinamensis*	Myristicaceae	SRB assay	Bark EO	[62]
			*IC*_50_ 9.41 μg/mL (HCT116), 16.93 μg/mL (HepG2), 20.64 μg/mL (HL-60), 29.52 μg/mL (B16–F10), 15.88 μg/mL (MCF-7) and 34.07 μg/mL (MRC-5).	
			Leaves EO	
			*IC*_50_ 26.70 μg/mL (HCT116), 7.07 μg/mL (HepG2), 22.76 μg/mL (HL-60), 18.80 μg/mL (B16–F10), 21.39 μg/mL (MCF-7) and 38.93 μg/mL (MRC-5)	

MTT(3-(4,5-dimethylthiazol-2-yl)-2,5-diphenyltetrazolium bromide). **** = statistically similar at 95% confidence level by Tukey’s test.

## Data Availability

Not applicable.
